# Bibliographical

**Published:** 1894-02

**Authors:** 


### Bibliographical.

Diseases and Injuries of the Teeth, Including Pathology and Treatment. A manual of Practical Dentistry for Students and Practitioners. By Morton Smale, M.R.C.S., L.S.A., L.D.S., and J. F. Colyer, L.R.C.P., M.R.C.S., L.D.S., of London.

In the preparation of this work the authors, in the preface, say: "In compiling this manual while an effort has been made to enunciate succinctly and plainly the principles underlying the art of dental surgery, yet in no case have the practical bearings of the subject been overlooked, and it is hoped in its present form that not only will dental students but general practitioners in medicine be able to follow its descriptions and directions with ease and profit."

A casual glance through this work warrants the statement that it presents a very good *resume*, very concisely and briefly stated, of all the particulars and details in common use in operative dentistry, bringing in collateral subjects that are necessary to be considered as a basis for the ordinary operations. An effort is made in a few particulars to adopt a better nomenclature; for example: Mandible for inferior maxilla; temporo mandibular articulation for temporo maxillary joint; stylo mandibular ligament, mandibular artery, nerve, etc.

It is not very apparent, nor is it here claimed, that there is any great advantage in the use of these terms over those usually employed; if, however, they are found to be better they will, of course, be adopted by the profession.

The Manipulative Department is very brief in many of its descriptions, which perhaps is well for the student, but for the practitioner fuller and more detailed statements would seem to be preferable.

Without entering into particulars, we cheerfully commend the work to the profession and to students in particular. The authors have done their work well. In subsequent editions, however, undoubtedly there will be changes made in some particulars. It is a work that ought to be in the library of every dentist and if every practitioner followed its directions closely, in the aggregate, an improvement would be made in dental practice.

